# *Chlorella sorokiniana*-Induced Activation and Maturation of Human Monocyte-Derived Dendritic Cells through NF-**κ**B and PI3K/MAPK Pathways

**DOI:** 10.1155/2012/735396

**Published:** 2012-11-25

**Authors:** Nien-Tzu Chou, Chieh-Fang Cheng, Hsin-Chieh Wu, Chin-Pen Lai, Li-Tsen Lin, I-Horng Pan, Ching-Huai Ko

**Affiliations:** Biomedical Technology and Device Research Laboratories, Industrial Technology Research Institute, Hsinchu 31040, Taiwan

## Abstract

*Chlorella sorokiniana* (CS) is a unicellular green alga. The extracts of *Chlorella* have been used as treatments for relieving hypertension and modulating immune response. The detailed mechanisms are not clear yet. In this study, we sought to study the molecular mechanisms for the polysaccharide fraction of CS-induced immune response. We pulsed dendritic cells (DCs) with CS and found that CS could maturate DCs. CS-maturated DC could activate naïve T cells and stimulate T-cell proliferation and IFN-**γ** secretion. Furthermore, CS activated PI3K and MAPKs signaling pathways in DCs by interacting with TLR4 receptor. These CS-activated signaling pathways could further activate NF-**κ**B and induce IL-12 production in DCs. This study provides molecular mechanisms for CS-induced DCs activation and immune response.

## 1. Introduction 

*Chlorella *is a genus of freshwater unicellular green algae. The extracts of *Chlorella* have been proposed as potential treatments for improving human health and wildly used as botanical foods. For example, *Chlorella* extracts were used as nutrition supplements in relieving hypertension and treatments for modulating human immune responses [1–4]. It was reported that *Chlorella* extracts can elicit various beneficial pharmacological effects against cancers [5], bacterial infections [6], and viral replication [7, 8]. From an earlier study, *Chlorella* extracts were shown to strongly increase the production of IFN-*γ* and IL-2 and activate Th1 cells to strengthen immune system and host defense [9]. Hasegawa et al. demonstrated the roles of *Chlorella* extracts in inducing IFN-*γ* and IL-2 mRNA expression and activating cell-mediated immunity [10]. 

DCs are professional antigen-presenting cells (APCs) and have unique ability in linking innate and adaptive immunity [11, 12]. Immature DCs are able to ingest antigens. Once activated, DCs go through a series of maturation processes that include migration to lymphoid tissues, downregulation of antigen uptake, upregulation of major histocompatibility complex (MHC) class II, costimulatory molecules (CD40, CD80, and CD86), and a specific maturation marker CD83 [13–15], and finally presenting antigenic peptides to T lymphocytes [16].

The MAPK families (p38, ERK, and JNK) were activated in response to a variety of cellular stress or stimuli, including oxidative stress, LPS and TNF-*α*, in all cell types indicated [17, 18]. 

The effects of CS on human DCs are not yet defined. In this study, we examined the molecular mechanisms of CS in the activation and maturation processes of human monocyte-derived DC. 

## 2. Materials and Methods 

### 2.1. Reagents

*Escherichia coli* LPS (L8274, *E. coli*), lipoteichoic acid (LTA; L2515, from *Staphylococcus aureus*), and H_2_O_2_ were purchased from Sigma Chemical Co. (St. Louis, MO, USA). Neutralization antibodies against toll-like receptor (TLR)-2 and TLR-4 were purchased from eBiosciences (San Diego, CA, USA). Helenalin, SB203580, PD98059, and JNK inhibitor II were purchased from Calbiochem (Darmstadt, Germany). LY294002 was purchased from LC Laboratories (Woburn, MA, USA). These inhibitors were dissolved in dimethyl sulfoxide (DMSO) from Sigma-Aldrich (St. Louis, MO, USA), and 0.1% (v/v) DMSO was used as negative control whenever indicated. The duration of pretreatment of these compounds (LY294002, helenalin, SB203580, PD98059, and JNK inhibitor II) on immature DCs before stimulation was 1 h unless indicated otherwise.

### 2.2. Preparation Polysaccharide Fraction of *Chlorella sorokiniana* (CS)

*Chlorella sorokiniana* is a commercially available product (International Cryptomonadales Biotechnology, Taiwan). Fifty grams of *Chlorella sorokiniana* powders were refluxed with 150 mL distilled water for 1 h. The extracts (polysaccharide fraction) were filtered through no. 5 filter paper (Toyo Roshi, Toyo, Japan) and vacuum concentrated at 60°C. The presence of LPS was detected by the chromogenic *Limulus* amebocyte lysate assay (Charles River Laboratories, Inc., Wilmington, MA, USA). The cytotoxicity of CS against normal cells (PBMC) was assessed by Alamar Blue assay (AbD Serotec, Oxford, UK) because of its low toxicity to normal cells [19, 20]. CS was not toxic to PBMC at the highest concentration tested (>100 *μ*g/mL) (data not shown).

### 2.3. Generation of Monocyte-Derived DC

All cell subsets were isolated from peripheral blood of healthy donors. Peripheral blood mononuclear cells (PBMCs, obtained from the Taiwan Blood Services Foundation, Taiwan) were isolated from heparinized whole blood by Ficoll/Isopaque/1.077 g/mL (Pharmacia, Freiburg, Germany) density gradient centrifugation (2800 rpm, 20 min, 22°C). CD14^+^ cells were purified by positive selection using anti-CD14^+^ microbeads with the MiniMACS system by following the manufacturer's instructions (Miltenyi Biotec, Auburn, CA, USA). The DC14^+^ cells were cultured at 1 × 10^6^/mL in 24-well plates (Costar, Cambridge, MA, USA) in RPMI 1640 with 10% fetal bovine serum (FBS), 2 mM L-glutamine, 25 mM HEPES, 100 U/mL penicillin, 100 U/mL streptomycin (Invitrogen, Carlsbad, CA, USA), granulocyte macrophage-colony stimulating factor (GM-CSF; 25 ng/mL), and IL-4 (25 ng/mL) (Peprotech, London, UK), and media were changed every 2-3 days. Human monocyte-derived DCs were harvested on day 6.

### 2.4. Cytokine Measurements

IL-12 and IFN-*γ* secreted from DCs or T cells were assayed with an enzyme-linked immunosorbent assay (ELISA) kit (R&D Systems, Minneapolis, MN, USA). The absorbance of the plate was detected by a SpectraMax M5 microplate reader (Molecular Devices, Sunnyvale, CA, USA) with input wavelength at 450–540 nm. The detecting limits of these ELISAs were 31.3 pg/mL for IL-12 and 15.6 pg/mL for IFN-*γ*.

### 2.5. Quantitative Reverse Transcription-Polymerase Chain Reaction (QRT-PCR)

For real-time RT-PCR analysis, the total RNA of cells was extracted by using the RNeasy Mini Kit (Qiagen). DNA was eliminated by Deoxyribonuclease I (Invitrogen). Briefly, the total RNA (0.8 *μ*g) of each sample was reversely transcribed with 0.5 *μ*g of oligo dT and 200 U SuperScript III RT (Invitrogen) in a 20 *μ*L reaction. PCR was carried out by StepOnePlus Real-Time PCR System (ABI) in a total volume of 25 *μ*L containing 0.5 mM of each primer, 1x Power SYBR Green PCR Master Mix (Life Technologies), and 50 ng cDNA. The quantification of the unknown samples was performed by StepOne Software, version 2.0 (ABI). GAPDH was amplified as a reference standard in each experiment. The primers used are as followed: IL12 p35 (F): GCCACAGGTCTGCATCCA, IL12 p35 (R): GACCTGGCGGGCTGAGTA, IL12 p40 (F): GGGGTGACGTGCGGAGCTGCT, IL12 p40 (R): TCTTGCCCTGGACCTGAACGC, GAPDH (F): GAAGGTGAAGGTCGGAGT, GAPDH (R): GAAGATGGTGATGGGATTTC.

### 2.6. Flow Cytometric Analysis

DCs were harvested, washed, and incubated with cold phosphate-buffered saline (PBS) (Invitrogen, Carlsbad, CA, USA). Cells were then incubated with dye-labeled monoclonal antibodies (mAbs) against target molecules (HLA-DR-FITC, CD83-PE, CD86-FITC, CD80-PE) (BD Biosciences PharMingen, San Diego, CA, USA) for 30 min on ice. Stained cells were then washed twice and resuspended in cold buffer and analyzed with a FACScan flow cytometry (Becton Dickinson, San Jose, CA, USA). More than 1 × 10^5^ cells were analyzed for each sample, and the results were processed by using WinMDI 2.8 software (Scripps Research Institute, La Jolla, CA, USA).

### 2.7. FITC-Labeled Dextran Uptake

Cultured DCs were washed twice and resuspended in 1 mL RPMI 1640 supplemented with 10% FBS, 2 mM L-glutamine, 100 U/mL penicillin, 100 U/mL streptomycin, and 25 mM HEPES. The cells were stimulated with CS (30 *μ*g/mL) for 48 h and then incubated with FITC-dextran (Sigma Chemical, St. Louis, MO, USA) (0.5 mg/mL) at 4°C or 37°C for 1 h. Afterward, the cells were washed thrice with ice-cold PBS and analyzed with a FACScan flow cytometry as described above.

### 2.8. Allogeneic Mixed Leukocyte Reaction (MLR)

An allogeneic MLR (allo-MLR) was performed by following a modified protocol [21]. After treated with CS, DCs were harvested and incubated with mitomycin-C (50 *μ*g/mL, Sigma Chemical, St. Louis, MO, USA) for 1 h at 37°C to inhibit cell proliferation. These cells were then washed three times with ice-cold PBS and plated into 96-well U-bottom culture plates (Corning, NY, USA) as stimulators for T cells.

CD3^+^ T cells for the allo MLR assay were obtained from allogeneic PBMC purified with MACS beads (Miltenyi Biotec, Auburn, CA, USA). The purity of isolated cells was >95% of CD3^+^ T cells as determined by flow cytometry. Purified CD3^+^ T cells (Responder cells) were added to 96-well U-bottom culture plates at 1 × 10^5^ cells/well. The final volume is 200 *μ*L/well, and the plates were incubated at 37°C in 5% CO_2_. After coculturing T cells and DC for 2 days, cell-free supernatants were collected, and secreted human IFN-*γ* was quantified by ELISA. T-cell proliferation was detected by Alamar Blue assay after being cocultured with DC for 5 days. The number of viable cells correlated with the magnitude of dye reduction and was quantified as percentage of alamarblue reduction. The percentage of alamarblue reduction (% reduction) is calculated according to the following formula:

(1) %  Reduction  (%  Viability)  =(O2×A1)−(O1×A2)(R1×N2)−(R2×N1)×100.

*O*_1_ and *O*_2_ are constants representing the molar extinction coefficient of oxidized alamarblue at 570 and 600 nm; *R*_1_ and *R*_2_ are molar extinction coefficients of reduced form of alamarblue at 570 and 600 nm, respectively. *A*_1_ and *A*_2_ represent absorbance of unknowns at 570 and 600 nm. *N*_1_ and *N*_2_ represent absorbance of negative controls at 570 and 600 nm, respectively. The values of % alamarblue reduction were calibrated with negative controls containing cell-free medium.

### 2.9. Neutralization Experiments

Human DCs were preincubated with 20 *μ*g/mL antibody against TLR-2 or TLR-4 (eBioscience, San Diego, CA, USA) for 1 h followed by LPS (1 *μ*g/mL), LTA (1 *μ*g/mL) (Sigma Chemical, St. Louis, MO, USA), or CS (30 *μ*g/mL) treatments for 24 h. The conditioned media were collected and analyzed for IL-12 secretion by ELISA [22].

### 2.10. Western Blotting

Total cellular lysates were prepared by using RIPA lysis buffer. Proteins in cell lysates (40 *μ*g) were separated on 10% SDS-polyacrylamide minigels and electrotransferred to a PVDF membrane by iBlot Dry Blotting System (Invitrogen, Carlsbad, CA, USA). Antibodies used in this study were as follows: total-JNK (rabbit pAb sc-571) (Santa Cruz Biotechnology, Santa Cruz, CA, USA), phospho-p38 (rabbit pAb #9211), total-p38 (rabbit pAb #9212), phospho-JNK (rabbit mAb #4668), phospho-p42/44 (rabbit pAb #9101), total-p42/44 (rabbit pAb #9102), phospho-AKT (rabbit mAb #2965), total-AKT (rabbit mAb #4691), *α*-tubulin (rabbit mAb #2125), and *β*-Actin (rabbit mAb #4970) (Cell Signaling Technology, Boston, MA, USA) [23, 24].

### 2.11. NF-*κ*B Assay

Human DCs were treated with CS (30 *μ*g/mL) or LPS (1 *μ*g/mL) for 15 min, 30 min, 2 h, or overnight. Nuclear proteins were extracted by using the Nuclear Extract Kit (Active Motif, Carlsbad, CA, USA), and protein concentration was determined by using the Bio-Rad Protein Assay (Bio-Rad Life Science Research, Hercules, CA, USA). For each assay, 5 *μ*g of extracted nuclear protein was used in a TransAM NF-*κ*B p65 ELISA kit (Active Motif, Carlsbad, CA, USA) according to the manufacturer's instruction. 

### 2.12. Statistical Analysis

Data were analyzed by Student's *t*-test and considered statistically significant if *P* < 0.05. All data were mean ± SEM of three independent experiments unless indicated otherwise. 

## 3. Results 

### 3.1. CS Induces Phenotypic Maturation and IL-12 Production of Human Monocyte-Derived DC by Activating NF-*κ*B

**Figure 1:**
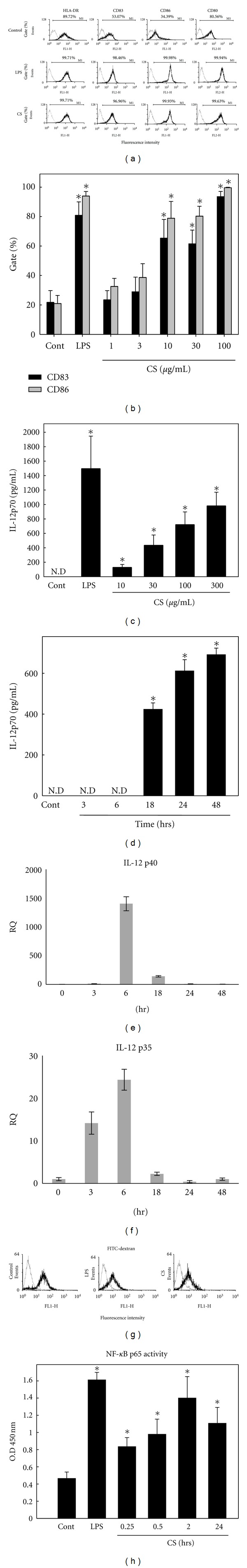
The effects of CS on DC maturation. (a) Human DCs were treated with CS (30 *μ*g/mL), LPS (1 *μ*g/mL), or medium alone for 48 h, and surface markers were analyzed by flow cytometry (dotted line, isotype control; solid line: specific mAb). The values shown were the percentage of gated cells (Gated %). (b) DCs were treated with CS at different concentrations, and the expressions of CD83 and CD86 were analyzed by flow cytometry (black bar: CD83; gray bar: CD86). LPS was used as positive control. (c) Human DCs were cultured in the presence of 1 *μ*g/mL LPS or various concentrations of CS for 48 h. IL-12 secretion was analyzed by ELISA after incubation. (d) The time-dependent effect of CS (30 *μ*g/mL) treatments on IL-12 secretion in DCs. IL-12 secretion was analyzed by ELISA. *P < 0.05 compared to control. N.D: nondetectable. ((e) and (f)) QRT-PCR analysis of IL-12 p35 and IL-12 p40. DCs were incubated in the presence of CS (30 *μ*g/mL) for 3, 6, 18, 24, and 48 h. Representative images of three independent experiments were shown here. Lane M: marker. (g) The effect of CS on DC endocytosis. Human PBMCs were cultured for 6 days, and monocytes were induced to differentiated DCs (refer to Section 2). Immature DCs were stimulated with medium alone, LPS (1 *μ*g/mL), or CS (30 *μ*g/mL) for 48 h, and then incubated with FITC-dextran (0.5 mg/mL) for 1 h at 4°C (dotted line) or 37°C (solid line). (h) Human monocyte-derived DC were incubated with CS (30 *μ*g/mL) for the indicated period of time. NF-*κ*B assay was described in Section 2. The binding activity of NF-*κ*B was shown as relative OD_450_ levels. LPS (1 *μ*g/mL) treatment for 2 h was used as positive control. *P < 0.05 compared to control.

LPS, a component of the Gram-negative bacterial cell membrane, was described as an inducer of DC activation and maturation [25]. Therefore, we use LPS as a positive control in this study. To determine whether CS can modulate the development of human DCs *in vitro*, we compared the phenotype of human DCs treated with or without CS for 48 h. We demonstrated that CS increased the expression of CD83, CD86, CD80, and MHC class II molecules on the cell surface of human DCs (Figures 1(a) and 1(b)). In order to test the properties of CS in stimulating IL-12 secretion in DCs, DCs were cultured in the presence of different amounts of CS. The production of IL-12 was dose dependently enhanced by CS treatment (Figure 1(c)). When treated with CS (30 *μ*g/mL) for different durations, human DCs secreted IL-12 time-dependently, and maximum secretion was observed within 18–48 h (Figure 1(d)). Therefore, the stimulatory effect of CS on IL-12 production in DCs was both dose and time dependent. To further examine whether CS could affect IL-12 expression, human DCs were treated with CS for indicated periods of time and assayed for IL-12 p35 and IL-12 p40 mRNA expression by QRT-PCR. Highest level of both IL-12 p35 and IL-12 p40 mRNA was expressed at 6 h posttreatment in human DCs (Figures 1(e) and 1(f)). 

Immature DCs capture and process antigens via endocytosis with high efficiency. Once entering the maturation processes, DCs lose their abilities to ingest and process antigens and become potent immunostimulatory APCs [26]. To study whether CS affected the endocytic capacities of DCs, we examined the uptake of FITC-labeled dextran by DCs. As LPS, CS reduced the endocytic capacities of DCs during DC maturation (Figure 1(g)).

LPS induced DC maturation through activating NF-*κ*B signaling [27]. To determine whether CS matured DC through a similar pathway, we monitored the translocation of NF-*κ*B into the nucleus induced by CS. DCs were cultured in the presence of CS for the indicated period of time, and nuclear extracts were collected and analyzed for NF-*κ*B binding activity using an ELISA-based assay. As shown in Figure 1(h), CS induced a rapid nuclear translocation of p65 and upregulated NF-*κ*B binding activity with maximum activity at 2 h poststimulation.

### 3.2. CS-Treated Human DCs Enhance T-Cell Activation

**Figure 2:**
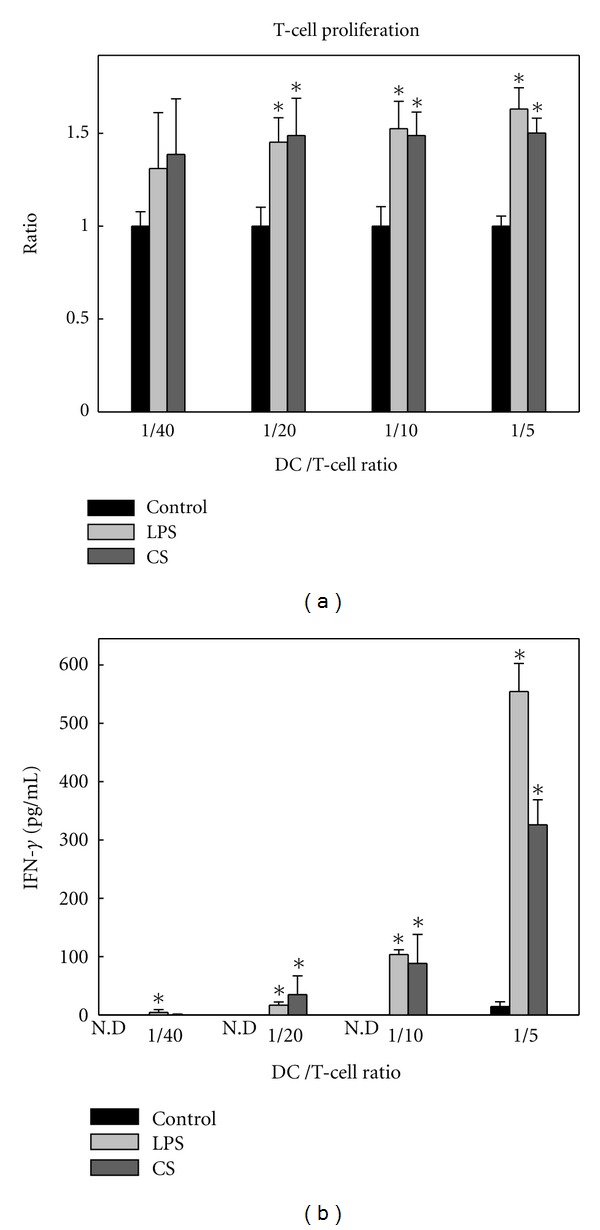
Allogeneic T-cell responses induced by CS-treated DCs. Immature DCs were stimulated with CS (30 *μ*g/mL) or LPS (1 *μ*g/mL) for 48 h. Allogeneic T cells (1 × 10^5^) were cocultured in 96-well U-bottom microplates with CS-treated DCs as described. (a) T-cell proliferation was determined by using an Alamar Blue assay 5 days post coculturing. Experimental groups were compared to corresponding control groups. (b) After 2 days of coculturing, the production of IFN-*γ* by T cells was analyzed by ELISA. Data were expressed as means ± SEM of triplicates from three independent experiments. *P < 0.05 compared to control. N.D: nondetectable.

Compared to immature DCs, mature DCs are capable of inducing allogeneic T-cell proliferation more efficiently [28]. Since CS could upregulate cell-surface markers and increase IL-12 production in DCs, we would like to study whether CS-induced DC maturation was sufficient in activating naïve T cells. Immature DCs were treated with LPS or CS and then cocultured with allogeneic naïve T cells. The results showed that CS-treated human DC enhanced T-cell activation, as evidenced by the secretion of IFN-*γ* by T cells (Figures 2(a) and 2(b)).

### 3.3. PI3K/AKT Pathway Acts Upstream of the MAPKs in CS-Stimulated DC

**Figure 3:**
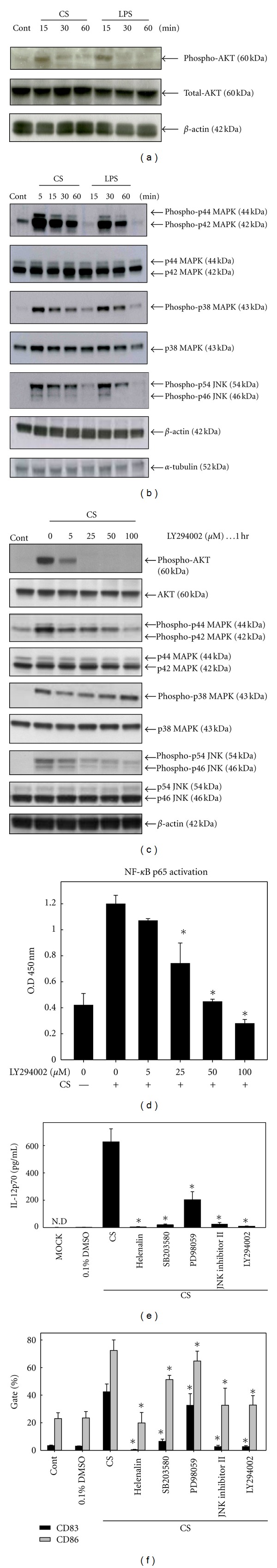
CS induces IL-12 secretion through PI3K/AKT and MAPKs pathways in DCs. ((a) and (b)) Time course of pAKT, p44/42 ERK, p38 MAPK, and p46/54 JNK phosphorylation in CS-stimulated DCs. Human DCs were treated with CS (30 *μ*g/mL), and cell lysates were collected at different time points. The level of MAPK phosphorylations was analyzed by Western blotting (*N* = 3). (c) Effect of PI3K inhibitor LY294002 on MAPKs in CS-stimulated DCs. Human DCs were pretreated with various concentration of LY294002 (5, 25, 50, and 100 *μ*M) for 1 h prior to CS (30 *μ*g/mL) for 15 min. Cell lysates were collected, and MAPK phosphorylations were analyzed (*N* = 3). (d) The PI3K inhibitor LY294002 repressed CS-induced NF-*κ*B p65 binding to DNA. Human DCs were pretreated with various concentration of LY294002 (5, 25, 50, and 100 *μ*M) for 1 h prior to 2 h stimulation by CS (30 *μ*g/mL). *P < 0.05 compared to CS alone. ((e) and (f)) Inhibitors against PI3K, NF-*κ*B, and MAPKs blocked CS-induced IL-12 secretion (e) and CD83, CD86 upregulation (f) (black bar: CD83; gray bar: CD86) in DC. Immature DCs were preincubated for 1 h with one of the following compounds: LY294002 (25 *μ*M), Helenalin (2.5 *μ*M), SB203580 (20 *μ*M), PD98059 (50 *μ*M), or JNK inhibitor II (20 *μ*M) and followed by CS (30 *μ*g/mL) stimulation for an additional 48 h. *P < 0.05 compared to CS alone. N.D: nondetectable.

We sought to study the effects of CS treatments in regulating intracellular signaling pathways in DCs. Human DCs were stimulated with CS, and the levels of MAPK phosphorylations were assessed by Western blotting. *β*-actin and *α*-tubulin were used for internal control. Our results showed that CS induced kinase phosphorylation of all MAPKs tested between 5 and 15 min after treatment (Figures 3(a) and 3(b)). To further study the roles of these signaling pathways in CS-induced DC maturation, we treated DC with chemical inhibitors against MAPKs. The doses of these inhibitors were determined based on cytotoxicity assay (see Supplemental Figure  1 available online at doi:10.1155/2012/735396). The maximum nontoxic concentration was used in our experiments. Pretreating cells with LY294002, a specific inhibitor of PI3K [29], resulted in blocking both CS-induced MAPKs phosphorylations and CS-induced AKT phosphorylation in DCs (Figure 3(c)). These data clearly illustrated that PI3K acted upstream of MAPKs in the CS-induced signaling cascades. In order to study the regulation of CS-induced NF-*κ*B activation, DCs were preincubated with LY294002. Our results showed that LY294002 abrogated CS-induced NF-*κ*B expression (Figure 3(d)). Next, we examined whether NF-*κ*B and MAPKs signaling was involved in the regulation of CS-stimulated DC activation and IL-12 secretion. Immature human DCs were pretreated with helenalin (a specific blocker of NF-*κ*B), SB203580 (a specific blocker of p38 MAPK), PD98059 (an inhibitor of the ERK pathway), JNK inhibitor II (an inhibitor of the JNK pathway), or LY294002 (an inhibitor of the PI3K pathway) for 1 h and subsequently stimulated with CS for 48 h. The production of IL-12 was quantified by ELISA. IL-12 secretion induced by CS was significantly abrogated by inhibitors against NF-*κ*B, PI3K, and MAPKs (Figure 3(e)). To further examine the involvement of NF-*κ*B and MAPKs in DC maturation, we blocked NF-*κ*B, PI3K, and MAPKs by helenalin and other MAPKs-specific inhibitors. All these inhibitors except PD98059, a specific inhibitor of ERK, significantly inhibited CS-induced upregulation of CD83 and CD86 in DCs (Figure 3(f)). These results showed that NF-*κ*B, PI3K, p38, and JNK played critical roles in DC maturation and IL-12 secretion. ERK was important for IL-12 secretion in DCs. However, the role of ERK in DC maturation was redundant and could be compensated by other signaling molecules.

### 3.4. CS-Induced TLR4 Signaling via the PI3K/AKT-MAPKs Pathway

**Figure 4:**
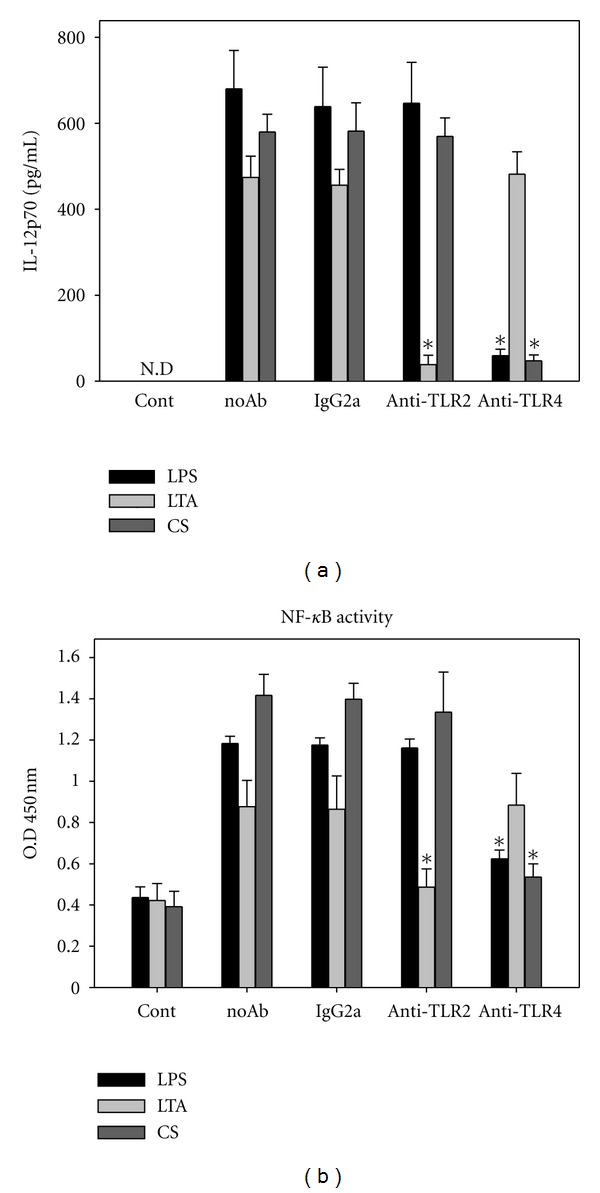
CS induces IL-12 expression through a TLR4-dependent ROS-regulated signaling pathway. (a) Anti-TLR-4 neutralizing antibody blocks CS-induced IL-12 secretion in DCs. DCs were preincubated with 20 *μ*g/mL anti-TLR-2, anti-TLR-4, or control IgG for 1 h prior to LPS (1 *μ*g/mL), LTA (1 *μ*g/mL), or CS (30 *μ*g/mL) treatments for 24 h. Conditioned media were collected for IL-12 detection. (b) NF-*κ*B binding activity was examined in DC treated with CS (30 *μ*g/mL) for 2 h with or without the pretreatments of neutralizing antibody against TLRs or control antibody for 1 h. The effects of neutralizing antibodies treatments were compared to corresponding control IgG treatments. *P < 0.05 compared to control antibodies. N.D: nondetectable.

Toll-like receptors (TLRs) are important pattern recognition receptors on cell surface of immune cells. LPS, Gram-negative bacterial cell membrane components, interact with TLR4. Gram-positive bacterial cell wall components such as lipoteichoic acid (LTA) are known ligands for TLR2 [30–32]. To determine whether these receptors were involved in CS-induced DC activation, neutralizing antibodies against TLR2 and TLR4 were added to DC 24 h prior to LPS, LTA, or CS treatments. As shown in Figure 4(a), anti-TLR4 neutralizing antibody significantly inhibited both CS- and LPS-induced IL-12 secretion, while anti-TLR2 neutralizing antibody significantly inhibited LTA-induced IL-12 secretion. In addition, anti-TLR4 neutralizing antibody also significantly reduced NF-*κ*B activation in CS-stimulated DCs (Figure 4(b)).

## 4. Discussion 

Algae are simple organisms, and their extracts can regulate immune responses in mammals [33, 34]. Among these algae, several species of *Chlorella* were reported to induce immune responses in human or mice [35–37]. In this study, we studied the effect of *Chlorella sorokiniana* polysaccharides extracts on human immune responses. It was reported that polysaccharides extracted from several origins can induce DC maturation and immune responses [38]. As other polysaccharide extracts, *Chlorella* polysaccharide fraction extracts can also maturate DC and activate immune response by inducing IL-12 secretion in DCs. 

IL-12 is important for activating natural killer cells (NKs) and inducing the differentiation of T helper cells toward Th1 cells. Th1 response can skew the immune system toward cellular immune response, which maximize the killing efficacy of macrophage, increase CD8^+^ T-cell proliferation, and activate natural killer cells [39]. In addition, Th1 response is important in fighting against virus infection and cancer [40]. 

Since NF-*κ*B binding site was found in the promoter region of both IL-12 p40 and p35, NF-*κ*B may involve in CS-induced IL-12 expression. Indeed, we observed that CS not only induced the expression of IL-12 in DC but also the activation of NF-*κ*B. Furthermore, blocking NF-*κ*B signaling in DCs reduced CS-induced IL-12 secretion. Therefore, NF-*κ*B signaling is required for CS-induced IL-12 expression in DCs. 

Signal transductions via the MAPKs play important roles in cellular responses including cell proliferation, differentiation, and survival [41]. We detected the activation of AKT and MAPK signaling pathways induced by CS in DC and verified the importance of AKT and MAPK signaling pathways induced by CS in DC activation. All signaling pathways tested were required for CS-induced IL-12 secretion in DC. However, ERK signaling pathway is not required for CS-induced DC maturation. The role of ERK signaling pathway in DC maturation is reported controversially. Lin et al. reported that ERK signaling pathway is required for IL-12 secretion in DC maturated by polysaccharide purified from *Ganoderma lucidum* [4]. However, ERK pathway was shown to differentiate DC towards tolerogenic DC [42]. One possible explanation is that the intensity and duration of signaling activation may be different based on stimuli, and different activation patterns of signaling pathways may result in various consequences. Further studies are required to clarify the importance of the intensity and duration of ERK phosphorylation in DC activation. 

TLRs are cell surface receptors that recognize structurally conserved molecules derived from microbes. The reported ligands of these TLRs range from bacterial cell surface LPS, lipoproteins, flagellin, viral DNA and dsRNA to host fibrinogen and heat shock proteins. Our results suggested that CS interacts with TLR4 and maturates DC through NF-*κ*B signaling pathway. Interestingly, TLR4 activates genes required for initiating adaptive immune responses when artificially ligated by antibodies, and NF-*κ*B signaling pathway was reported involved in this process [43]. Consistent with previous publications, we demonstrated the importance of TLR4 signaling in activating immune response. Furthermore, we identified CS extracts as novel ligands for TLR4. In order to rule out the contamination of endotoxin LPS in CS, we detected LPS level in CS samples. We found that there was no detectable level of endotoxin (<0.30 endotoxin units/mL) in CS samples (data not shown). Therefore, our results suggested that CS maturates DC by binding to TLR4 receptor.

## 5. Conclusion

**Figure 5:**
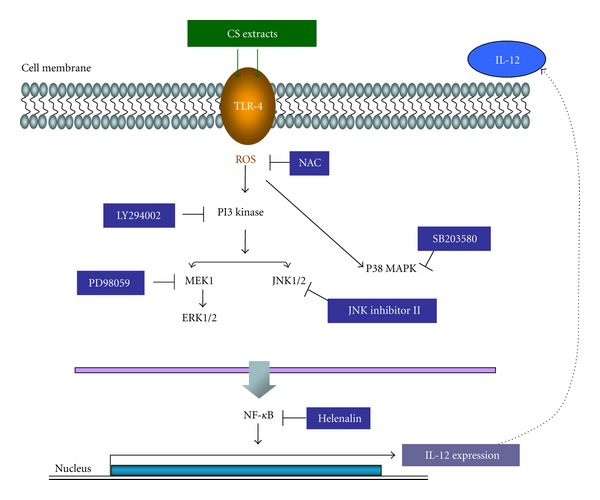
Model of CS-mediated signaling pathways in regulating IL-12 p70 expression in DC.

In summary, our study provides potential molecular mechanisms for CS-induced DC maturation and T-cell activation. These mechanisms are illustrated in a schematic figure (Figure 5). In addition, our study also provided theoretical basis for studying the effects of CS on immune responses in animal models and clinically.

## Supplementary Material

To determine the concentration for MAPK inhibitors used in this study, the dose-dependent cytotoxicity of these MAPK inhibitors on DC were tested. The MAPK inhibitors tested includes Helenalin, SB203580, PD98059, JNK inhibitor II, and LY294002. The maximum nontoxic concentration was determined in this experiment and was used throughout the study.

## Abbreviations

CS: The polysaccharide fraction of *Chlorella sorokiniana *
MTT: (3-(4,5-dimethylthiazol-2-yl)-5-(3-carboxyMethoxy-phenyl)-2-(4-sulfophenyl)-2H-tetrazolium
MAPK: Mitogen-activated kinase cascade
ERK: Extracellular regulated kinase
JNK: Jun N-terminal kinase
PBMC: Peripheral blood mononuclear cell
DCs: Dendritic cells
HMDC: Human monocyte-derived DC
APCs: Antigen-presenting cells
LPS: Lipopolysaccharides
LTA: Lipoteichoic acid
MHC class II: Major histocompatibility complex class II
PI3K: Phosphoinositide-3-OH kinase
IFN-*γ*: Interferon-*γ*
TLR-4: Toll-like receptor 4
TLR-2: Toll-like receptor 2
IL-12: Interleukin-12
GM-CSF: Granulocyte macrophage-colony stimulating factor
IL-4: Interleukin-4
NF-*κ*B: Nuclear factor *κ* B
NAC: N-acetyl-cysteine
ELISA: Enzyme-linked immunosorbent assay
GAPDH: Glyceraldehyde 3-phosphate dehydrogenase
Allo-MLR: Allogeneic mixed leukocyte reaction.

## References

[1] Merchant RE, Andre CA (2001). A review of recent clinical trials of the nutritional supplement *Chlorella pyrenoidosa* in the treatment of fibromyalgia, hypertension, and ulcerative colitis. *Alternative Therapies in Health and Medicine*.

[2] Yang F, Shi Y, Sheng J, Hu Q (2006). *In vivo* immunomodulatory activity of polysaccharides derived from *Chlorella pyrenoidosa*. *European Food Research and Technology*.

[3] An HJ, Rim HK, Jeong HJ, Hong SH, Um JY, Kim HM (2010). Hot water extracts of *Chlorella vulgaris* improve immune function in protein-deficient weanling mice and immune cells. *Immunopharmacology and Immunotoxicology*.

[4] Lin YL, Liang YC, Lee SS, Chiang BL (2005). Polysaccharide purified from *Ganoderma lucidum* induced activation and maturation of human monocyte-derived dendritic cells by the NF-*κ*B and p38 mitogen-activated protein kinase pathways. *Journal of Leukocyte Biology*.

[5] Tanaka K, Yamada A, Noda K (1998). A novel glycoprotein obtained from *Chlorella vulgaris* strain CK22 shows antimetastatic immunopotentiation. *Cancer Immunology Immunotherapy*.

[6] Queiroz MLS, Rodrigues APO, Bincoletto C, Figueirêdo CAV, Malacrida S (2003). Protective effects of *Chlorella vulgaris* in lead-exposed mice infected with *Listeria monocytogenes*. *International Immunopharmacology*.

[7] Ibusuki K, Minamishima Y (1990). Effect of *Chlorella vulgaris* extracts on murine cytomegalovirus infections. *Natural Immunity and Cell Growth Regulation*.

[8] Santoyo S, Plaza M, Jaime L, Ibañez E, Reglero G, Señorans FJ (2010). Pressurized liquid extraction as an alternative process to obtain antiviral agents from the edible microalga *Chlorella vulgaris*. *Journal of Agricultural and Food Chemistry*.

[9] An HJ, Rim HK, Lee JH (2008). Effect of *Chlorella vulgaris* on immune-enhancement and cytokine production *in vivo* and *in vitro*. *Food Science and Biotechnology*.

[10] Hasegawa T, Kimura Y, Hiromatsu K (1997). Effect of hot water extract of *Chlorella vulgaris* on cytokine expression patterns in mice with murine acquired immunodeficiency syndrome after infection with *Listeria monocytogenes*. *Immunopharmacology*.

[11] Banchereau J, Steinman RM (1998). Dendritic cells and the control of immunity. *Nature*.

[12] Banchereau J, Briere F, Caux C (2000). Immunobiology of dendritic cells. *Annual Review of Immunology*.

[13] Mellman I, Steinman RM (2001). Dendritic cells: specialized and regulated antigen processing machines. *Cell*.

[14] Sallusto F, Cella M, Danieli C, Lanzavecchia A (1995). Dendritic cells use macropinocytosis and the mannose receptor to concentrate macromolecules in the major histocompatibility complex class II compartment: downregulation by cytokines and bacterial products. *Journal of Experimental Medicine*.

[15] Zhou LJ, Tedder TF (1996). CD14+ blood monocytes can differentiate into functionally mature CD83+ dendritic cells. *Proceedings of the National Academy of Sciences of the United States of America*.

[16] Lanzavecchia A, Sallusto F (2001). Regulation of T cell immunity by dendritic cells. *Cell*.

[17] Bunyard P, Handley M, Pollara G (2003). Ribotoxic stress activates p38 and JNK kinases and modulates the antigen-presenting activity of dendritic cells. *Molecular Immunology*.

[18] Ardeshna KM, Pizzey AR, Devereux S, Khwaja A (2000). The PI3 kinase, p38 SAP kinase, and NF-*κ*b signal transduction pathways are involved in the survival and maturation of lipopolysaccharide-stimulated human monocyte-derived dendritic cells. *Blood*.

[19] Ahmed SA, Gogal RM, Walsh JE (1994). A new rapid and simple non-radioactive assay to monitor and determine the proliferation of lymphocytes: an alternative to [^3^H]thymidine incorporation assay. *Journal of Immunological Methods*.

[20] Zhi-Jun Y, Sriranganathan N, Vaught T, Arastu SK, Ahmed SA (1997). A dye-based lymphocyte proliferation assay that permits multiple immunological analyses: mRNA, cytogenetic, apoptosis, and immunophenotyping studies. *Journal of Immunological Methods*.

[21] Kato K, Cantwell MJ, Sharma S, Kipps TJ (1998). Gene transfer of CD40-ligand induces autologous immune recognition of chronic lymphocytic leukemia B cells. *The Journal of Clinical Investigation*.

[22] Zughaier SM (2011). Neisseria meningitidis capsular polysaccharides induce inflammatory responses via TLR2 and TLR4-MD-2. *Journal of Leukocyte Biology*.

[23] Ko CH, Shen SC, Lin HY (2002). Flavanones structure-related inhibition on TPA-induced tumor promotion through suppression of extracellular signal-regulated protein kinases: involvement of prostaglandin E2 in anti-promotive process. *Journal of Cellular Physiology*.

[24] Ko CH, Shen SC, Lee TJF, Chen YC (2005). Myricetin inhibits matrix metalloproteinase 2 protein expression and enzyme activity in colorectal carcinoma cells. *Molecular Cancer Therapeutics*.

[25] De Smedt T, Pajak B, Muraille E (1996). Regulation of dendritic cell numbers and maturation by lipopolysaccharide *in vivo*. *Journal of Experimental Medicine*.

[26] Banchereau J, Steinman RM (1998). Dendritic cells and the control of immunity. *Nature*.

[27] Rescigno M, Martino M, Sutherland CL, Gold MR, Ricciardi-Castagnoli P (1998). Dendritic cell survival and maturation are regulated by different signaling pathways. *Journal of Experimental Medicine*.

[28] Cella M, Sallusto F, Lanzavecchia A (1997). Origin, maturation and antigen presenting function of dendritic cells. *Current Opinion in Immunology*.

[29] Vlahos CJ, Matter WF, Hui KY, Brown RF (1994). A specific inhibitor of phosphatidylinositol 3-kinase, 2-(4-morpholinyl)-8-phenyl-4H-1-benzopyran-4-one (LY294002). *The Journal of Biological Chemistry*.

[30] Akira S, Takeda K (2004). Toll-like receptor signalling. *Nature Reviews Immunology*.

[31] Visintin A, Latz E, Monks BG, Espevik T, Golenbock DT (2003). Lysines 128 and 132 enable lipopolysaccharide binding to MD-2, leading to toll-like receptor-4 aggregation and signal transduction. *The Journal of Biological Chemistry*.

[32] Sato S, Nomura F, Kawai T (2000). Synergy and cross-tolerance between Toll-like receptor (TLR) 2- and TLR4-mediated signaling pathways. *The Journal of Immunology*.

[33] Halperin SA, Smith B, Nolan C, Shay J, Kralovec J (2003). Safety and immunoenhancing effect of a *Chlorella*-derived dietary supplement in healthy adults undergoing influenza vaccination: randomized, double-blind, placebo-controlled trial. *Canadian Medical Association Journal*.

[34] Balachandran P, Pugh ND, Ma G, Pasco DS (2006). Toll-like receptor 2-dependent activation of monocytes by *Spirulina* polysaccharide and its immune enhancing action in mice. *International Immunopharmacology*.

[35] Guzmán S, Gato A, Lamela M, Freire-Garabal M, Calleja JM (2003). Anti-inflammatory and immunomodulatory activities of polysaccharide from *Chlorella stigmatophora* and *Phaeodactylum tricornutum*. *Phytotherapy Research*.

[36] Hasegawa T, Kimura Y, Hiromatsu K (1997). Effect of hot water extract of *Chlorella vulgaris* on cytokine expression patterns in mice with murine acquired immunodeficiency syndrome after infection with *Listeria monocytogenes*. *Immunopharmacology*.

[37] Rim H-K, An H-J, Lee J-H, Seo M-J (2008). Effect of *Chlorella vulgaris* on immune-enhancement and cytokine production *in vivo* and *in vitro*. *Food Science and Biotechnology*.

[38] Chan WK, Law HKL, Lin ZB, Lau YL, Chan GCF (2007). Response of human dendritic cells to different immunomodulatory polysaccharides derived from mushroom and barley. *International Immunology*.

[39] Takeda K, Tsutsui H, Yoshimoto T (1998). Defective NK cell activity and Th1 response in IL-18-deficient mice. *Immunity*.

[40] Shurin MR, Lu L, Kalinski P, Stewart-Akers AM, Lotze MT (1999). Th1/Th2 balance in cancer, transplantation and pregnancy. *Springer Seminars in Immunopathology*.

[41] Brunet A, Datta SR, Greenberg ME (2001). Transcription-dependent and -independent control of neuronal survival by the PI3K-Akt signaling pathway. *Current Opinion in Neurobiology*.

[42] Boggiatto PM, Jie F, Ghosh M (2009). Altered dendritic cell phenotype in response to *Leishmania amazonensis* amastigote infection is mediated by MAP kinase, ERK. *The American Journal of Pathology*.

[43] Medzhitov R, Preston-Hurlburt P, Janeway CA (1997). A human homologue of the *Drosophila* toll protein signals activation of adaptive immunity. *Nature*.

