# Supplementary material for: Chlorella sorokiniana-Induced Activation and Maturation of Human Monocyte-Derived Dendritic Cells through NF-κB and PI3K/MAPK Pathways
Source: Evid Based Complement Alternat Med. 2012 Nov 25;2012:735396. doi: 10.1155/2012/735396 (PMC3523612; doi:10.1155/2012/735396)
Supplement: Supplementary file 1 — To determine the concentration for MAPK inhibitors used in this study, the dose-dependent cytotoxicity of these MAPK inhibitors on DC were tested. The MAPK inhibitors tested includes Helenalin, SB203580, PD98059, JNK inhibitor II, and LY294002. The maximum nontoxic concentration was determined in this experiment and was used throughout the study. [file 735396.f1.pdf]

Supplemental Figure

Chou et al., Supplemental Figure 1

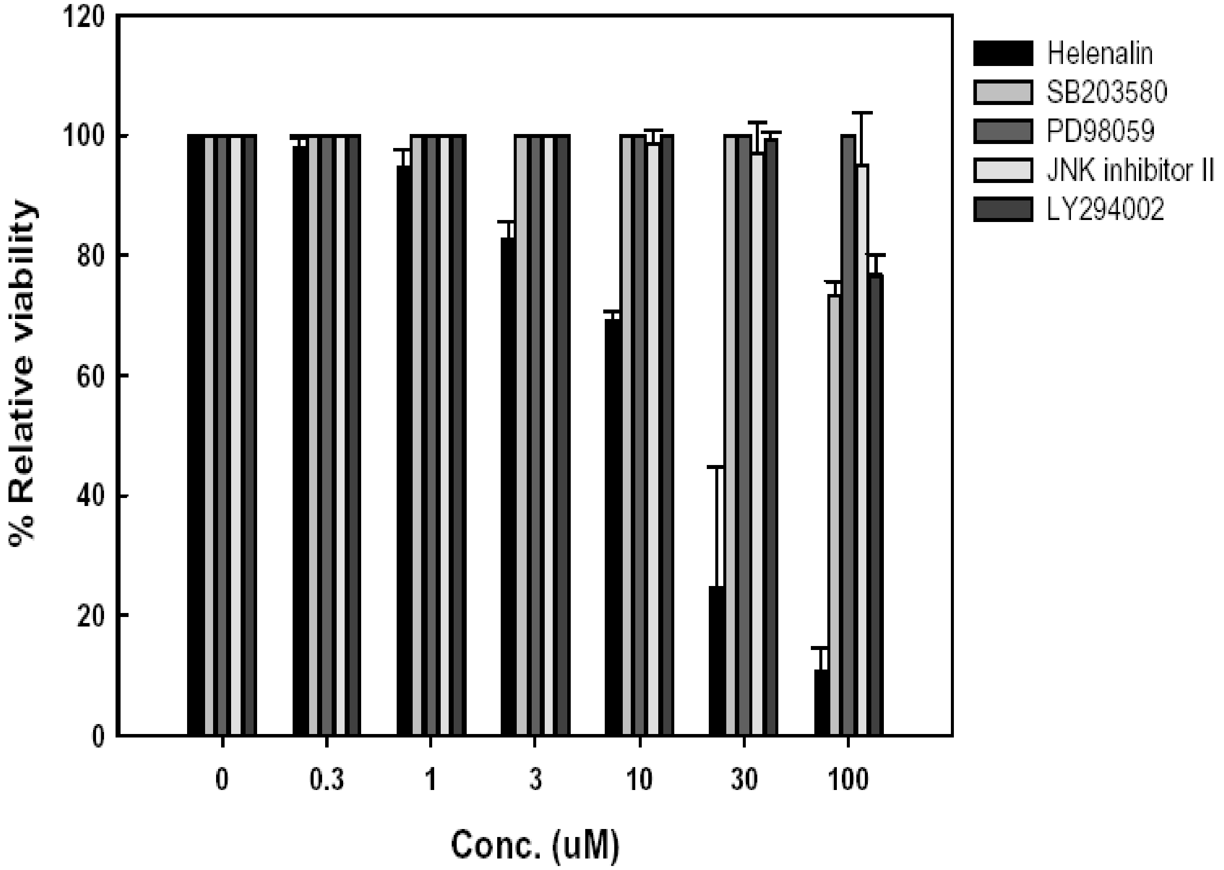

Suppplemental Figure 1. Dose-dependent cytotoxicity of MAPK inhibitors. PBMCs treated with different doses of MAPK inhibitors for 72hr, and followed by alamarblue treatment for 24hr (N=3).
